# Diabetes exacerbates retinal oxidative stress, inflammation, and microvascular degeneration in spontaneously hypertensive rats

**Published:** 2012-06-02

**Authors:** Islam N. Mohamed, Sahar A. Soliman, Ahmed Alhusban, Suraporn Matragoon, Bindu A. Pillai, Ahmed A. Elmarkaby, Azza B. El-Remessy

**Affiliations:** 1 Program in Clinical and Experimental Therapeutics, College of Pharmacy, University of Georgia, Augusta, GA; 2 Vision Discovery Institute, Georgia Health Science University, Augusta, GA; 3 Department of Oral Biology, Georgia Health Science University, Augusta, GA; 4 Charlie Norwood Veterans Affairs Medical Center, Augusta, GA

## Abstract

**Purpose:**

Hypertension and diabetes are known risk factors for retinal microvascular damage. However, the combined effects of diabetes with early and established stages of hypertension on retinal microvascular degeneration remain incompletely understood.

**Methods:**

Male spontaneously hypertensive rats (SHR) were compared to SHR with streptozotocin-induced diabetes (SHR+D) for 6 or 10 weeks and Wistar rats as controls.

**Results:**

Hypertension alone (the SHR group) or in combination with diabetes (the SHR+D group) for 6 weeks induced additive increases in total retinal cell death, compared to the Wistar controls. This increase was associated with significant increases in phosphorylated-Jun N-terminal kinase (pJNK) activation, phosphorylated-Akt inhibition, plasma and retinal lipid peroxides, and soluble intracellular adhesion molecule-1 (sICAM-1) levels. After 10 weeks, a similar trend was still observed in retinal nitrotyrosine, nuclear factor kappaB p65, and tumor necrosis factor-α expression, associated with exacerbated pJNK activation and formation of acellular capillaries.

**Conclusions:**

In conclusion, combining diabetes and hypertension-potentiated retinal oxidative/inflammatory stress promoted imbalance between the JNK stress and survival Akt pathways resulting in accelerated retinal cell death and acellular capillary formation.

## Introduction

Hypertension has been identified as an independent risk factor for developing retinopathy. The results of the Beaver Dam Eye 15-year cumulative study in non-diabetic subjects showed that uncontrolled blood pressure is associated with increased incidence of retinopathy [1]. Early phases of hypertensive retinopathy are characterized by increased retinal microvascular changes, highlighted by generalized retinal arteriolar narrowing due to increased vascular tone [2]. However, the exact mechanism behind hypertensive retinopathy is not fully understood. Several mechanisms have been proposed, including oxidative stress [3], inflammation [4], and resulting endothelial dysfunction [5].

Diabetic retinopathy is the second major cause of blindness in working adults in the United States [6]. Accelerated death of retinal capillary cells leading to vaso-obliteration and acellular-occluded capillaries are well defined histopathological changes of diabetic retinopathy in clinical and experimental models [7-9]. We and others have previously established diabetes-induced peroxynitrite formation and inflammation as the major molecular mechanisms responsible for retinal endothelial dysfunction and vascular cell death [10-18].

Diabetes and hypertension are common comorbid conditions that have been identified as independent risk factors for the development of endothelial dysfunction [19,20]. Clinical evidence indicates a beneficial effect of lowering blood pressure on the progression of diabetic retinopathy [21-23]. However, the effects of early and established stages of hypertension combined with diabetes on the development of retinopathy and possible contribution to retinal microvascular degeneration remain incompletely understood. The aim of the current study was to examine the effects of early and established hypertension alone or in combination with diabetes—as known risk factors for vascular damage—on development of retinopathy and retinal microvascular degeneration. Our results identify key molecular mechanisms involved such as imbalance between the stress Jun N-terminal kinase (JNK) and survival protein kinase B (PKB/Akt) pathways and increased systemic and retinal oxidative/inflammatory stress, resulting in increased retinal cell death and exacerbated acellular capillary formation.

## Methods

### Animal preparation

All of the animal studies were conducted in accordance with the Association for Research in Vision and Ophthalmology (ARVO) Statement for the Use of Animals in Ophthalmic and Vision Research and the Charlie Norwood Veterans’ Affairs Medical Center Animal Care and Use Committee. Six-week-old male spontaneously hypertensive rats (SHR) were randomly assigned to an SHR or diabetic SHR (SHR+D) group. Normal Wistar Kyoto (W) rats served as control. Diabetes was induced in the SHR by a single intravenous injection of streptozotocin (STZ, 60 mg/kg). Detection of glucose in urine and blood glucose levels >13.9 mmol/l indicated diabetes. Rats were supplemented with insulin pellets (subcutaneously) to prevent ketoacidosis. Rats were weighed weekly, and blood glucose measurements were taken from a tail vein using a glucometer. As shown in (Table 1), animals injected with STZ had significant increases in blood glucose levels and decreases in bodyweight compared with the control W group and the SHR group. Animal groups were deeply anesthetized for terminal sacrifice using intraperitoneal injection of ketamine/xylazine mixture (48 mg/kg and 6.4 mg/kg, respectively; Phoenix Pharmaceuticals, St. Joseph, MO) after 6 or 10 weeks of diabetes induction.

**Table 1:** Effects of STZ-induced diabetes on bodyweight and blood glucose levels in rat groups.

**Group**	**n**	**End weight (g)**	**End blood glucose mmoles/l**
**6-weeks**			
W	12	316±9	6.9±0.4
SHR	12	314±7	6.5±0.37
SHR+D	12	265±13*	27±3*
**10-weeks**			
W	6	380±8	7.7±0.8
SHR	6	372±7	8±0.5
SHR+D	6	248±13*	28.7±3*

Rats were rendered diabetic by a single streptozotocin injection (60 mg/kg) in freshly prepared 10 mmol/l sodium citrate buffer, pH 4.5. Data are mean±SEM *Significant difference versus other groups at p<0.05.

### Evaluation of cell death in rat retina

Quantitative cell death was assessed with terminal deoxynucleotidyl transferase dUTP nick-end labeling (TUNEL) assay in flatmounted retinas using immunoperoxidase staining (ApopTag-Peroxidase; Millipore Corporation, Billerica, MA), as described previously by our group [24]. Briefly, whole, flat-mounted samples were stained with 3-amino-9-ethylcarbazole (Sigma, St. Louis, MO) following the manufacturer’s instructions. The total number of TUNEL-positive cells per retina was counted using light microscopy. Appropriate negative and positive controls were used to assess proper reactivity.

### Detection of lipid peroxides

Levels of lipid peroxides (malondialdehyde, MDA) were assayed using thiobarbituric acid reactive substances (TBARS) as described before by our group [24]. Briefly, plasma or retinal homogenate were acidified with 20% acetic acid, 8% sodium dodecyl sulfate, and thiobarbituric acid at 95 °C for 60 min, and then the reaction was cooled down on ice. The samples were centrifuged, the supernatant was extracted with n-butanol and pyridine (15:1, respectively), and the absorbance of the organic solvent layer was measured colorimetrically at 532 nm. The results were compared to an external standard (tetramethoxypropane). The Bradford assay (Bio-Rad, Hercules, CA) was also performed to determine the protein concentration of the retinal lysate. The lipid peroxide level was expressed in µM MDA/mg total protein.

### Detection of systemic soluble intracellular adhesion molecule-1

Plasma soluble intracellular adhesion molecule-1 (sICAM-1) levels were measured as an indicator of systemic inflammation using the quantitative sandwich enzyme-linked immunoassay (ELISA) technique (R&D systems, Minneapolis, MN). Briefly, plasma samples from all groups were pipetted into microplate wells precoated with a monoclonal antibody specific for rat sICAM-1. The color produced after an enzyme-linked polyclonal antibody was added was specific for rat sICAM-1, and the substrate solution was measured colorimetrically at 450 nm.

### Detection of retinal peroxynitrite formation

Slot-blot analysis was used to indirectly measure peroxynitrite levels with its footprint nitrotyrosine (NY). As described previously [24], 5 μg of retinal homogenate from rat samples were immobilized onto a nitrocellulose membrane. After blocking, the membrane was reacted with antinitrotyrosine polyclonal antibody (Calbiochem, Millipore) followed by its relative secondary antibody, and the optical densities of various samples were compared to those of the controls [24].

### Western blot analysis

Retinas were lysed in modified radioimmunoprecipitation assay (RIPA) buffer (Millipore, Billerica, MA) for 30 min on ice. Insoluble material was removed by centrifugation at 14,000× g at 4 °C for 30 min. About 30–50 µg of total protein were boiled in 6× Laemmli sample buffer, separated on a 10%–12% sodium dodecyl sulfate–polyacrylamide gel by electrophoresis, transferred to nitrocellulose membranes, and then reacted with specific primary and secondary antibodies respectively. The primary antibodies for Akt, phosphorylated-Akt (pAkt), c-JNKs, phosphorylated-JNKs (pJNKs), total nuclear factor kappaB (NFκB) p65 (all from Cell signaling, Danvers, MA), and tumor necrosis factor-α (TNF-α; Novus Biologicals, Littleton, CO) were used and detected using a horseradish peroxidase-conjugated antibody and enhanced chemiluminescence (Thermoscientific, Rockford, IL). Nitrocellulose membranes were subsequently scanned, and band intensities were quantified using (Alpa Innotech, Protein simple, Santa Clara, CA) imaging and densitometry software.

### Isolation of retinal vasculature

Retinal vasculatures were isolated as described previously [25]. Briefly, freshly enucleated eyes were fixed with 2% paraformaldehyde overnight. Retina cups were dissected, washed in phosphate-buffered saline, and then incubated with 3% Difco-Trypsin 250 (BD Biosciences, San Jose, CA) in 25 mM Tris buffer, pH 8, at 37 °C for 2 h. Vitreous and nonvascular cells were gently removed from the vasculature. The vasculature was then soaked in several washes of 5% Triton X-100 to get rid of the neural retina. The transparent vasculature was laid out on slides and used for acellular capillary examination.

### Determination of degenerated (acellular) capillaries

Retinal vasculature sections were stained with periodic acid-Schiff and hematoxylin (PASH). Acellular capillary counts were quantified under microscope (400×) in a masked manner. Acellular capillaries were identified as capillary-sized blood vessel tubes having no nuclei anywhere along their length. The number of acellular capillaries were counted in eight different fields of the mid-retina and then averaged together, indicating the number/high power field of each image.

### Data analysis

Results were expressed as mean±standard error of the mean (SEM). Differences among experimental groups were evaluated with ANOVA, and the significance of differences between groups was assessed with the post hoc test (Fisher’s protected least significant difference) when indicated. Significance was defined as p<0.05.

## Results

### Early stage hypertension induces and diabetes exacerbates retinal cell death

**Figure 1:**
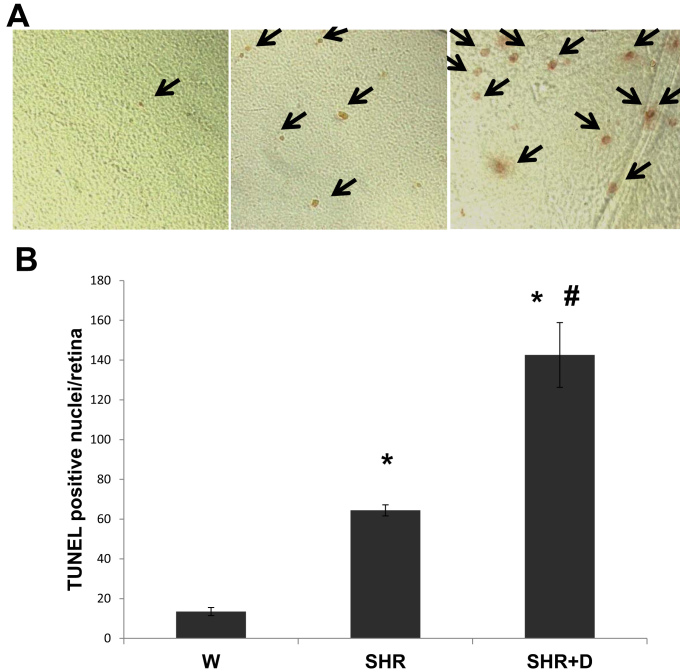
Early stage hypertension and diabetes exacerbates retinal cell death. **A**: Flat-mounted retinal samples showing increased total retinal cell death, as indicated with TUNEL-positive nuclei using immunoperoxidase staining, in early stage spontaneous hypertensive rats (SHR) which was exacerbated in diabetic spontaneous hypertensive rats (SHR+D) compared to control wistar group (W). **B**: Statistical analysis of the number of TUNEL-positive cells showed a 4.7 fold (64.4±2.8) increase in the number of apoptotic cells in the SHR alone group and 10.6 fold (142.6±16.3) increase in the combined SHR+D group when compared to the W control group (13.5±2; n=5–7, *p<0.001). There was also a 5.8 fold significant difference between the SHR+D and SHR groups (#p=0.002).

Apoptosis begins early in diabetes and likely contributes to neuronal death and later capillary obliteration, which are important features of retinopathy [26-28]. Here, we examined whether early stages of hypertension alone or combined with diabetes can cause retinal cell death. After the rats underwent 6 weeks of hypertension with or without diabetes, quantitative assessment of retinal cell death in the flatmounted retinas showed that the SHR alone group had a 4.7-fold increase in the total number of TUNEL positive nuclei/retina that was further increased 10.6 fold in the combined SHR+D group, compared to the W control group (Figure 1A,B).

### Early stage hypertension and diabetes stimulate activation of Jun N-terminal kinase stress pathway

**Figure 2:**
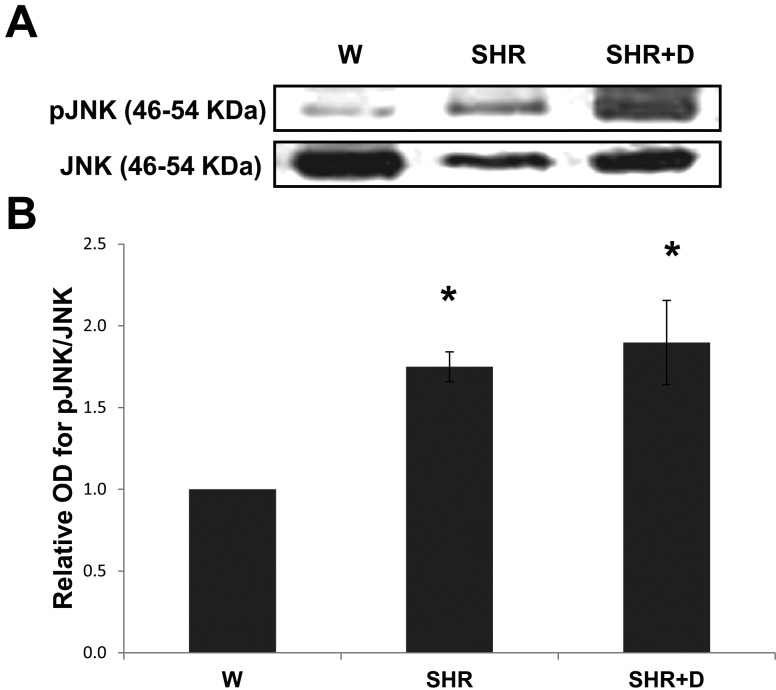
Early stage hypertension and diabetes stimulate activation of the Jun N-terminal kinase (JNK) stress pathway. **A**: Representative image for western blot analysis of retinal phosphorylated- pJNK protein expression in early stage spontaneous hypertensive rats (SHR) and diabetic spontaneous hypertensive rats (SHR+D) compared to control wistar group (W). **B**: Statistical analysis showing that activation of pJNK was 1.75 fold higher in the SHR group that was increased to 1.9 fold in the combined SHR+D group relative to the control W group (n=3-4, * p<0.05).

We sought to assess whether this early increase in retinal cell death was a result of activation of stress pathways. The SHR alone and combined SHR+D groups induced phosphorylation of the stress JNK protein 1.7 and 1.9 fold, respectively, relative to the control W group (Figure 2A,B).

### Early stage hypertension and diabetes impair Akt survival pathway

**Figure 3:**
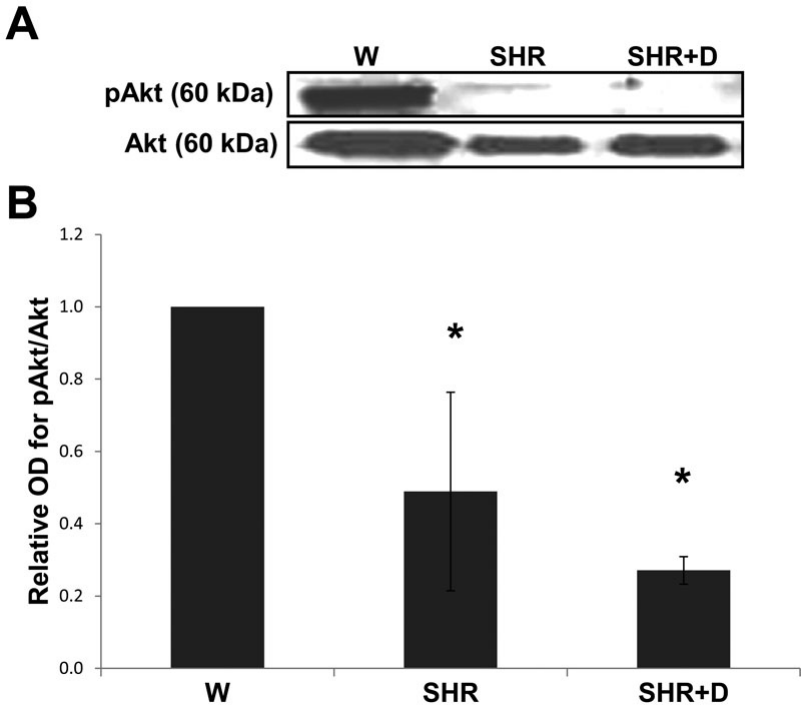
Early stage hypertension and diabetes suppress the Akt survival pathway. **A**: Representative image for western blot analysis of retinal phosphorylated-AKt (AKt) protein expression in early stage spontaneous hypertensive rats (SHR) and diabetic spontaneous hypertensive rats (SHR+D) compared to control wistar group (W). **B**: Statistical analysis showing that activation of pAkt was inhibited by 0.5 fold in the SHR group that was increased to 0.7 fold in the combined SHR+D group relative to the control W group (n=3-4, *p<0.05).

In addition, activation of the JNK stress pathway was mirrored by the inhibition of the Akt survival pathway. Akt protein phosphorylation was inhibited 0.5-fold in the SHR group and 0.7 fold in the combined SHR+D group relative to the control W group (Figure 3A,B).

### Early stage hypertension causes and diabetes exacerbates oxidative stress and inflammation

**Figure 4:**
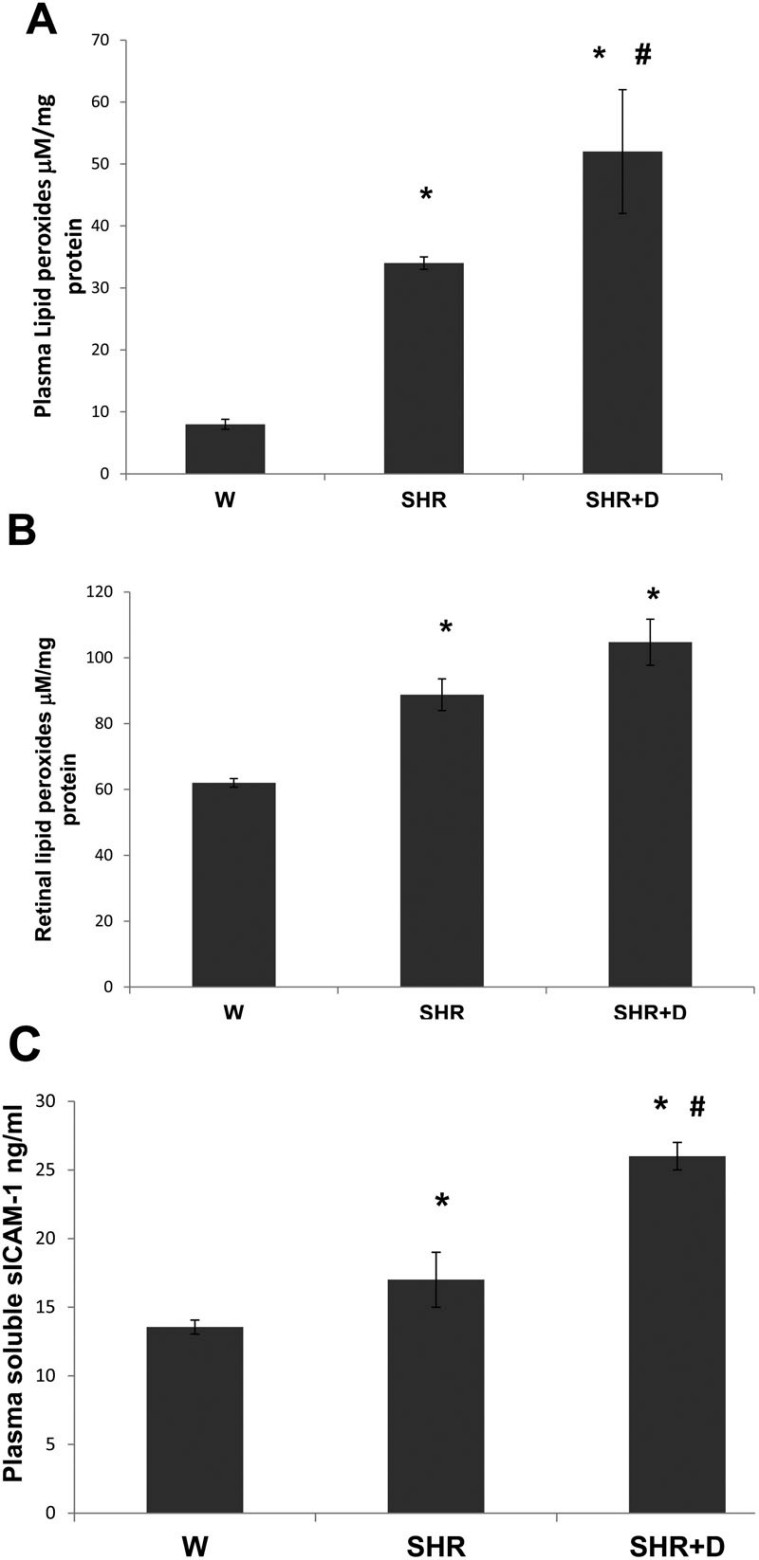
Early stage hypertension and diabetes exacerbate oxidative stress and inflammation. **A**: Plasma thiobarbituric acid reactive substances (TBARS) levels were higher in the spontaneously hypertensive rats (SHR) alone group by 4.5 fold, whereas the combined diabetic spontaneous hypertensive rats (SHR+D) group plasma levels were higher by 6.5 fold when compared to control W group (n=6, *p<0.05). There was a 1.53 fold difference in plasma TBARS levels between the SHR+D and SHR groups (#p<0.05). **B**: Retinal lipid peroxides were higher in the SHR group by 1.43 fold and in the SHR+D group by 1.69 fold (n=4, *p<0.05). **C**: Plasma sICAM-1 were higher in the SHR alone group by 1.25 fold and in the combined SHR+D group by 1.9 fold when compared to control W group (n=6, *p<0.05). There was also a 0.65 fold difference in sICAM-1 plasma levels between the SHR+D and SHR groups (p<0.05).

Hypertension and diabetes are known pro-oxidative stress and proinflammatory conditions. Therefore, we examined plasma and retinal lipid peroxide and plasma sICAM-1 levels as markers of oxidative and inflammatory stress. The plasma and retinal lipid peroxide levels increased 4.5 and 1.4 fold in the SHR alone group whereas the combined SHR+D group levels were higher by 6.5 and 1.7 fold, respectively, compared to the control W group (Figure 4A,B). In parallel, an 1.25 fold increase in plasma sICAM-1 levels was also detected in the SHR alone group and a 1.9 fold increase in the combined SHR+D group when compared to the control W group (Figure 4C).

### Established hypertension and diabetes maintain exacerbated retinal nitrative stress

**Figure 5:**
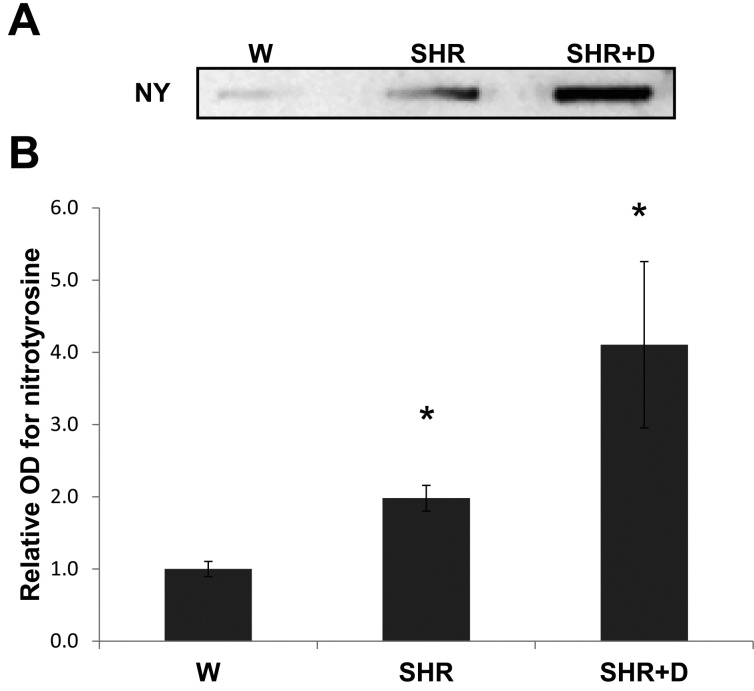
Established hypertension alone or in combination with diabetes stimulates retinal peroxynitrite formation. **A**: Representative image shows slot blot analysis of retinal nitrotyrosine (NY) levels in various groups. **B**: Retinal NY levels were higher in the SHR and SHR+D groups by 1.98 fold and 4.1 fold, respectively, relative to the control W group (n=4, *p<0.05).

We next examined the effects of later stage hypertension with or without diabetes (after 10 weeks of duration). A similar trend of increased oxidative stress was still observed through increased levels of retinal NY, the footprint for peroxynitrite, implicating retinal endothelial dysfunction [13]. Retinal NY levels in the SHR alone group increased 1.98 fold and 4.1 fold in the SHR+D group relative to the control W group (Figure 5A,B).

### Established hypertension and diabetes maintain exacerbated retinal inflammation

**Figure 6:**
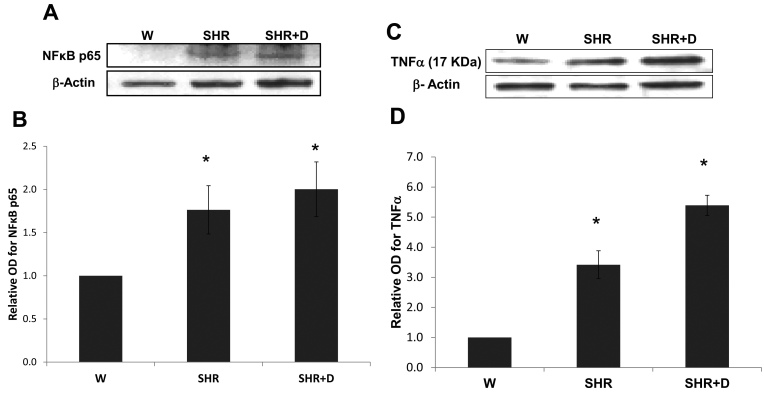
Established hypertension alone or in combination with diabetes stimulates retinal inflammation. **A**: Representative image for western blot analysis of retinal nuclear factor kappaB p65 (NFkB p65) protein expression in the established stage of spontaneous hypertensive rats (SHR) and diabetic spontaneous hypertensive rats (SHR+D) compared to control wistar group (W). **B**: Representative image for western blot analysis of retinal tumor necrosis factor aplha (TNF-α) protein expression in the established stage of SHR and SHR+D compared to control wistar group (W; n=4, *p<0.05). **C**: Representative image shows results of western blot analysis of retinal TNF-α protein expression. **D**: Statistical analysis showed that TNF-α protein expression was higher in the SHR and SHR+D groups by 3.4 and 5.4 fold, respectively, relative to the control W group (n=4, *p<0.05).

Similarly, late stage hypertension with or without diabetes also sustained in retinal inflammation evidenced by increased NFκB p65 protein expression. Retinal inflammation was 1.76 and twofold higher in the SHR alone and SHR+D groups, respectively, relative to the control W group (Figure 6A,B). NFκB is known to induce the transcription of different proinflammatory cytokines, including TNF-α, which is essential for inducing apoptosis in retinal neuronal and endothelial cells [29]. TNF-α protein expression was 3.4 fold higher in the SHR alone group relative to the control W group, and increased 5.4 fold in the combined SHR+D group (Figure 6C,D).

### Established hypertension maintains and diabetes exacerbates activation of Jun N-terminal kinase stress pathway

**Figure 7:**
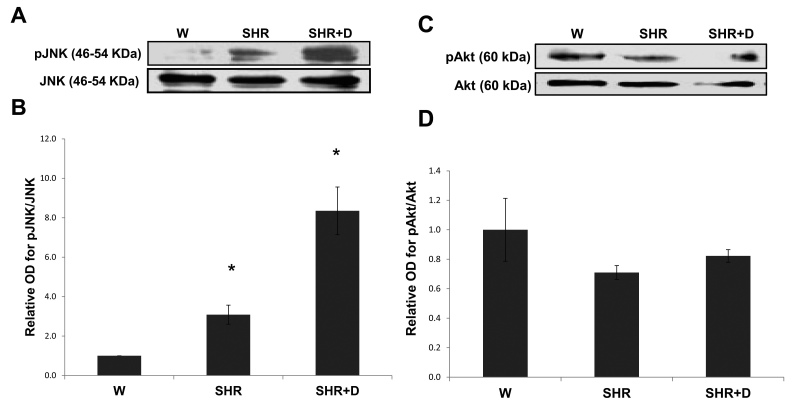
Established hypertension alone or in combination with diabetes stimulates JNK stress pathway and impairs the survival Akt pathway. **A**: Representative image for western blot analysis of retinal phosphorylated-Jun N-terminal kinase (pJNK) protein expression in the established stage of spontaneous hypertensive rats (SHR) and diabetic spontaneous hypertensive rats (SHR+D) compared to control wistar group (W). **B**: Statistical analysis showed that activation of pJNK was higher by threefold in the SHR group that was increased to 8.35 fold in the combined SHR+D group relative to the control W group (n=3, *p<0.05). **C**: Representative image for western blot analysis of retinal phosphorylated-AKt (AKt) protein expression in the established stage of SHR and SHR+D compared to W. **D**: Statistical analysis showing that activation of pAkt tends to be inhibited by 0.3 fold in the SHR group and 0.4 fold in the combined SHR+D group relative to the control W group (n=3, p=0.19 and 0.26, respectively).

As shown in (Figure 7A,B), activation of the JNK stress pathway was further exacerbated at 10 weeks 3 and 8.35 fold in the SHR and combined SHR+D groups, respectively. We next evaluated activation of the survival pathway Akt. Results showed a trend toward decreased expression of total Akt levels and decreased Akt phosphorylation in the SHR alone and SHR+D groups yet did not reach statistical significance in either group (Figure 7C,D).

### Established hypertension causes and diabetes exacerbates retinal vascular degeneration

**Figure 8:**
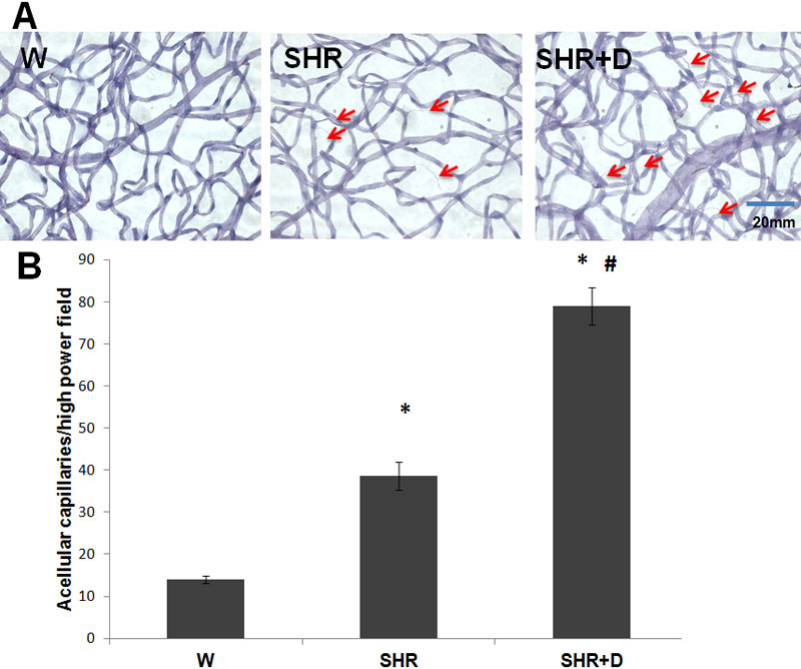
Established hypertension causes and diabetes exacerbates retinal microvascular degeneration. **A**: Representative images for retinal trypsin digests stained with periodic acid-Schiff and hematoxylin (PASH) to assess the development of acellular capillaries in the established stage of spontaneous hypertensive rats (SHR) and diabetic spontaneous hypertensive rats (SHR+D) compared to control wistar group (W). Acellular capillaries were defined as capillary-sized blood vessel tubes having no nuclei anywhere along their length (arrows). **B**: Statistical analysis for the average number of acellular capillaries per group, showed higher numbers in the SHR and SHR+D groups by 2.8 and 5.6 folds, respectively, when compared to control W group (n=4–5; *p<0.05). The SHR+D group was higher than the SHR group by twofolds (#p<0.05). The SHR+D group was higher than the SHR group by twofold (#p<0.05).

We finally evaluated the effects of 10 weeks of hypertension alone or in combination with diabetes on retinal microvascular degeneration, the hallmark of retinopathy, as assessed by acellular capillary formation. Retinal tryptic digests stained with periodic acid-Schiff and hematoxylin showed higher numbers of acellular capillaries in the SHR group by 2.8 fold that was exacerbated in the SHR+D group to 5.64 fold relative to the control W group (Figure 8A,B).

## Discussion

There is growing evidence supporting the notion that endothelial dysfunction and inflammation are common events driving the development of various retinal microvascular changes in patients with hypertension, diabetes, and other metabolic disorders [30,31]. However, the synergistic impact of these commonly coexisting conditions on the development of retinopathy has not been fully elucidated. Our current study aimed to model the early and established detrimental effects of combining hypertension with diabetes on the development of retinopathy and retinal microvascular degeneration using diabetic spontaneously hypertensive rats, an established model for studying hypertension.

Clinical evidence highlights the role of oxidative stress and endothelial dysfunction as previously indicated by increased levels of lipid peroxidation and platelet activation in patients with hypertension-related microvascular changes when compared with patients with or without early signs of retinopathy [3,30]. Our results showed the SHR group demonstrated significant increases in systemic and retinal lipid peroxides as early as 6 weeks of hypertension, which was maintained through increased retinal NY levels, the footprint of peroxynitrite, at 10 weeks. In fact, our previous studies established NY as a legitimate marker for retinal endothelial dysfunction as a result of endothelial nitric oxide synthase uncoupling and decreased bioavailability of nitric oxide [13,14]. These findings lend further support to other studies showing increased oxidative stress and nitrotyrosine levels in prehypertensive SHR [32,33].

Inflammation has been proposed as an essential partner for oxidative stress in the development of hypertensive retinopathy [5,34]. Our data demonstrated a significant increase in the plasma sICAM-1 levels in the early stage of hypertension and a significant upregulation of the NFκB p65/TNF-α pathway in the established stage. In accordance, an association between increased levels of high-sensitivity C-reactive protein, a marker of systemic low-grade inflammation, and the extent of microvascular complications in hypertensive patients was clinically observed [4]. In addition, a previous report has also shown a significant increase in NFκB p65 expression in prehypertensive SHR and a trend toward the upregulation of retinal ICAM-1 expression and macrophage infiltration in established hypertensive rats that did not reach statistical significance [34].

Hypertension frequently coexists with diabetes [19,20]. Major clinical studies such as the UK Prospective Diabetes Study Group and others have shown beneficial effects of tight blood pressure control and antihypertensive renin-angiotensin system blockers on prevention and progression of retinopathy in diabetic patients [22,35,36]. Therefore, we examined the combined effects of diabetes on hypertension on observed levels of oxidative stress and inflammation. Our results demonstrated that the combined SHR+D group had exacerbated oxidative stress and inflammation at early stage and established levels, as indicated by increased systemic and retinal lipid peroxide, plasma sICAM, retinal NY, NFκB p65, and TNF-α levels. In line with our findings, a similar cohort of studies by Lopes de Faria et al. [32,34] has also shown additive increases in superoxide anion production, NY formation, in addition to higher NFκB p65 and ICAM-1 expression, resulting in increased retinal macrophage infiltration in prehypertensive and hypertensive diabetic SHR, respectively. Most of the aggravating effects of diabetes on hypertensive retinas were corrected by reestablishing the redox balanced state and inducible nitric oxide synthase suppression using an exogenous superoxide dismutase mimetic [33] or angiotensin receptor blocker [37,38], indicating the convergence between hyperglycemia and the rennin-angiotensin system on oxidative stress pathways that may also lead to associated increases in inflammation.

In response to diabetes, apoptosis of retinal neuroglial cells begins as early as 4 weeks [26] and likely contributes to neuroglial inflammation culminating in capillary obliteration, retinopathy lesions, and increased formation of non-perfused acellular capillaries [7,27,28]. In agreement, our results showed that hypertension alone or in combination with diabetes accelerated retinal cell death as indicated by TUNEL-positive cells as early as 6 weeks of duration. The majority of apoptotic cells may be of neuronal origin at that time point [26]. In addition, Lopes de Faria et al. have shown increased total retinal DNA damage after 3 weeks of hypertension only or combined with diabetes [34,38] and exacerbated levels of TUNEL positive retinal cells in diabetic SHR that were mainly in neuronal and glial cells after 12 weeks [37].

Retinal oxidative/nitrative and inflammatory stress has been closely associated with activation of p38 and JNK–mitogen-activated protein kinases, resulting in apoptosis [10,12,39]. Here, we report that early stage hypertension (SHR) activated JNK phosphorylation after just 6 weeks compared to the W controls. These effects were paralleled with a reduction in the activation of the Akt survival pathway. Combining hypertension with diabetes (SHR+D) increased JNK activation and further impaired Akt phosphorylation compared to the W control group. Moreover, JNK activation was further exacerbated at 10 weeks in addition to maintaining the trend toward impaired expression and activation of Akt. Despite previous observations that significant increases in acellular capillary formation are usually observed after 6 months of diabetes [8,40,41], interestingly, the observed JNK/Akt imbalance coincided with significant increases in acellular capillary formation in the SHR alone group or in combination with diabetes at 10 weeks (Figure 8). Retinal microvascular cell death can occur as a result of increased leukostasis, direct biochemical insults within microvascular cells, or indirectly by neuroglial cells (reviewed in [27]). One of the limitations of our current study is that we did not dissect whether the observed imbalance between JNK and Akt pathways and resulting increases in apoptosis occurred predominantly in microvascular cells. Hence, further studies are warranted for such investigations. Nevertheless, the proapoptotic role of JNK activation has been demonstrated in inducing total retinal, pericyte, and microvascular cell death in vivo and in vitro [39,42-44]. We have also previously demonstrated a role for oxidative stress in altering the Akt/p38 MAP kinase balance, hence promoting endothelial cell death via peroxynitrite-mediated PI3-kinase tyrosine nitration in models of hyperglycemia and ischemic retinopathy [12,45]. Accelerated retinal capillary dropout, vascular tortuosity, and vascular leakage were previously reported in obese SHR starting at 12 weeks of age, which became more striking at 10 months [46]. Another group has also showed aggravation of the number of acellular capillaries and arteriolar microthromboses in SHR+D compared to diabetic W rats, which was mitigated by treatment with aminoguanidine, an advanced glycation product formation inhibitor, but after 26 weeks of diabetes [47]. Of note, to the best of our knowledge, we are the first group to report an imbalance between the retinal stress JNK and survival Akt pathways in either the SHR alone model or in its combination with diabetes in vivo.

In summary, these results suggest potentiating effects between systemic hypertension and diabetes as independent risk factors for retinopathy. This emphasizes the importance of addressing different cardiovascular risk factors in a holistic approach for improving management and prevention of retinopathy. Further studies are warranted to screen the protective effects of inhibiting JNK stress pathway activation and restoring Akt survival signals in the development of retinopathy in hypertension with or without diabetes and to dissect their causative role in retinal microvascular degeneration.
